# On matrix-analytic Markov modulated models for evolution: application potential and future directions required to advance the field

**DOI:** 10.3389/fsysb.2026.1906462

**Published:** 2026-10-07

**Authors:** Małgorzata M. O’Reilly, Barbara R. Holland

**Affiliations:** 1 School of Natural Sciences (Mathematics), University of Tasmania, Hobart, TAS, Australia; 2 Australian Research Council Centre of Excellence for Plant Success, University of Tasmania, Hobart, TAS, Australia

**Keywords:** approximate dynamic programming, evolution, likelihood, Markov modulated models, matrix-analytic methods, simulation

## Abstract

Markov modulated (MM) models in the theory of matrix-analytic methods (MAMs) are a class of probabilistic models whose key feature is an underlying Markov environment that drives the evolution of some complex and unpredictable system. Our goal in this paper is to emphasise their great potential for understanding evolution and the need for close multidisciplinary collaborations between probabilists and evolutionary biologists to advance the field. We give an overview of the fundamental MM models in MAMs, namely quasi-birth-and-death processes (QBDs) and stochastic fluid models (SFMs), in which the fluctuations in the level variable are modulated by fluctuations in the phase variable. We illustrate their application potential in evolution through an example of phylogenetic inference, state a general tree likelihood formula applicable to a wide range of MM models, and discuss future directions required to advance the theory of MM models as well as in the applications of the existing theory to evolutionary contexts.

## 1 Introduction

We consider matrix-analytic *Markov modulated* models studied in Soewongsono et al. (2025), in which the construction of the model involves a Markov process used to describe the underlying environment that drives (modulates) the evolution of a system in time, such as a gene family, species tree, or a trait that evolves on the tree. Our view is that these classes of models enable powerful methodologies for the analysis of evolutionary processes and have immense, not fully explored potential for understanding evolution. Furthermore, advances in these models through close multidisciplinary collaborations between probabilists and evolutionary biologists could open the door to novel solution methods and insights.

The key advantages of such Markov modulated (MM) models in the context of modelling evolution are: they can describe the evolutionary processes in a natural and meaningful way; they can be readily translated into simulations to produce insightful numerical outputs; their analysis can lead to closed-form/recursive expressions for key metrics including the likelihood; their parameters can be obtained through numerical optimisation of the likelihood or by applying prior biological knowledge.

In our discussion, we focus on two fundamental, closely-related (Aksamit et al., 2025) classes of Markov modulated (MM) models: Quasi-Birth-and-Death processes (QBDs); and Stochastic Fluid Models (SFMs); each recording state (X(t),φ(t)) of a system at a continuous time t with a state consisting of a *level* variable X(t) and a *phase* variable φ(t) . The difference between these two models is that the level variable is discrete in the QBDs and continuous in the SFMs, with different applications to real-world processes. In these Markov models, the fluctuations in the level variable are determined (driven/modulated) by fluctuations in the phase variable through model parameters. Understanding QBDs and SFMs is fundamental in the construction and analysis of many classes of MM models with level-dependent or time-varying parameters. The key advantage of these models is that “complicated and tedious calculations are circumvented” by the use of matrix-analytic methods which are based on matrix manipulations (Hobolth et al., 2019).

In SFMs, phase is used to describe an underlying environment modelled as a finite-state continuous-time Markov chain (CTMC) {φ(t):t≥0} with generator $T=\left[T_{\varphi \varphi^{\prime }}\right]$ and level is used to model some continuous quantity modulated by the Markov chain and the so-called fluid rates $c_{\varphi \left(t\right)}=dX\left(t\right)/dt$ which could be positive/negative/zero. As an example in the context of modelling evolution, level could be some continuous trait of the species, e.g. body mass, which increases/decreases/remains constant depending on the phase which could represent some aspect of the environment the species inhabits or a discrete trait, e.g. oviparity vs. vivaparity. SFMs–which to our knowledge have not yet been applied in an evolutionary context–could provide an alternative to methods such as SURFACE (Mahler, 2013), a generalisation of an OU process (Butler and King, 2004), which attempts to detect regime shifts on a phylogeny where a continuous trait may have a different mean under different regimes.

In QBDs, level is used to model some discrete quantity such as the number of genes in a gene-family, or the number of individuals in a population. The QBD itself is a a continuous-time Markov chain with generator $Q=\left[q_{\left(n,\varphi \right)\left(n^{\prime },\varphi^{\prime }\right)}\right]$ , and skip-free structure so that transitions $\left(n,\varphi \right)\to \left(n^{\prime },\varphi^{\prime }\right)$ are only possible to levels $n^{\prime }=n-1,n,n+1$ . This results in a convenient structure of the generator that is partitioned into much smaller, finite-size blocks $Q^{\left[n.n^{\prime }\right]}$ containing transition rates between the neighbouring levels, which enables efficient numerical analysis based on fast algorithms. QBDs could have application in population genetics where, e.g. the level variable could indicate the number of individuals with a particular allele and the phase could represent environments where the allele is under stronger or weaker selection. MM models with transitions $\left(n,\varphi \right)\to \left(n^{\prime },\varphi^{\prime }\right)$ not limited to only levels $n^{\prime }=n-1,n,n+1$ have also been studied using techniques from the theory of QBDs. For example, Joyner and Fralix (2016) developed results for CTMCs in which transition are permitted to all $n^{\prime }\leq n$ . Also, Baele et al. (2021) proposed Markov-modulated CTMCs to incorporate time variability of evolutionary processes, and implemented them in BEAST based on the results for finite-state CTMCs. This idea could be extended to QBDs.

We note that the level variable X(t) in QBDs/SFMs can be used to model some discrete/continuous property of an evolving system. Phase variable φ(t) , which drives the fluctuations in the level variable, can be used in a mechanistic manner, or by numerically fitting to data. Mechanistic models which seek to describe underlying processes in a manner that explains how system components interact (Casadevall and Fang, 2009), have been successfully used to model evolutionary processes in many contexts (Teufel et al., 2014; Stark et al., 2017; Diao, 2023; Soewongsono, 2023). In some cases the phase variable could have a direct interpretation. For example, in Diao et al. (2022) and Stark et al. (2017) the phase variable modelled the extent to which members of gene family were redundant in the sense that the functions they performed were also performed by other members of the gene family. In other cases the phase component of the model could be fit numerically as a way to generalise exponential waiting times. This latter approach was taken by Soewongsono et al. (2025) to fit waiting times until speciation events.

Crucially, the MM models allow for modelling a wide range of distributions of time spent in a particular state. This is useful as there are numerous applications in the study of evolution where it is desirable to generalize exponential waiting times (Hössjer et al., 2021). This feature is enabled through an application of Phase-Type (PH) distributions which record time till absorption in an absorbing Markov chain (Latouche and Ramaswami, 1996). PH can be used to approximate positive-valued distributions (Asmussen et al., 1996), and it can be easily fit to many empirical distributions using existing tools (Hobolth et al., 2019; Marshall et al., 2005). Some existing evolutionary models can be seen as special cases of PH models. For example, the covarion model of DNA substitution (Tuffley and Steel, 1998) can be thought of as a mechanistic PH model where the phase corresponds to a site being able or unable to accept a substitution. Similarly, in the extension by Galtier (2001) the underlying phase represents the rate class of the site. In the context of birth-death models, Soewongsono et al. (2022) found that PH distributed times to speciation gave superior fits to empirical species trees in terms of their balance. Hobolth et al. (2019) demonstrated that important quantities in coalescent models can often be expressed in terms of PH, and provided a unified theory and analysis for a number of coalescent models. We refer the reader to their work for the details of the theory of PH methods and the applications of PH in population genetics.

## 2 Discussion

### 2.1 Theory and tools for the analysis of the Markov modulated (MM) models

We summarise the key literature containing theoretical results and tools for the stationary (long-run) and transient (functions of time) analysis of the MM models. The SFMs and QBDs are two fundamental *classes* of models (with many types of models analysed) in the rich theory of matrix-analytic-methods pioneered by Neuts (1981). Examples of application potential of the QBDs to evolutionary processes were given in Soewongsono et al. (2025), including modelling species trees, gene trees, and their reconciliation.

For the overview of theoretical results and algorithms for the analysis of the SFMs we refer the reader to (Ahn and Ramaswami, 2003; Da Silva Soares, 2005; Bean et al., 2005b; a, 2008; Bean and O’Reilly, 2013) and references within. Transient analysis considers densities ∂/∂xP(X(t)≤x,φ(t)=φ) (since the level is a continuous variable); and probabilities P(X(t)=0,φ(t)=φ) for models with a boundary at level zero. Stationary analysis involves their limits as t→∞ , assuming that the process is stable with a negative long-run mean drift $\mu =\sum_{\varphi }c_{\varphi }\left(\lim_{t\to \infty }P\left(\varphi \left(t\right)=\varphi \right)\right)<0$ .

For an overview of results for the analysis of QBDs we refer the reader to Neuts (1981); Ramaswami (1997); Joyner and Fralix (2016); Latouche and Ramaswami (1996); Phung-Duc et al. (2010); He (2014) and references therein. Transient analysis involves probabilities P(X(t)=n,φ(t)=φ) while stationary analysis involves their limits as t→∞ under sufficient stability conditions.

We note that a wide range of tools are available for the numerical analysis of the MM models. BuTools (Horváth and Telek, 2017) contains a library of functions based on the computational methods for the analysis of QBDs and other Markovian models, for implementation in Matlab, Mathematica and Python. BuTools includes functions to handle Phase-Type distributions. SMCSolver (Bini et al., 2006; Pérez et al., 2008) contains MATLAB functions to compute the stationary distribution of the QBDs. MAMSolver (Riska and Smirni, 2002) provides implementation of matrix-analytic algorithms in Matlab and C++ for the QBDs.

Finally, we would like to mention Markovian Binary Trees (MBTs) introduced in Kontoleon (2006), which is a class of Markov models that has a tree-like structure, and it also falls in the category of the QBDs. For further analysis of the MBTs the reader is referred to Hautphenne et al. (2008), Hautphenne et al. (2011); Hautphenne (2015); Soewongsono et al. (2025). As Soewongsono et al. (2025) point out, some later-introduced evolutionary models (Maddison et al., 2007; FitzJohn, 2012) are in fact sub-types (special cases) of more general MBTs, and so the existing theory of the MBTs and the QBDs could be readily applied to derive many useful stationary and transient quantities of interest, and by doing so, considerably extend the current analysis. This potential has been illustrated in detail by Soewongsono et al. (2025), who considered quantities such as probability of species becoming extinct, probability of a species surviving, probability of observing $n_{L}$ tips on the left subtree on the tree with n tips, or the likelihood of observing a species-gene tree. The analysis was obtained through efficient matrix-analytic techniques from the literature of QBDs and MBTs, involving differential equations and recursive algorithms written in a convenient, matrix form.

### 2.2 Application example: Phylogenetic inference and future directions

Now, consider a tree T that started at time $t_{0}$ on which N branching events have occurred at times $t_{1},\ldots ,t_{N}$ , with $t_{0}<t_{1}<\ldots <t=t_{N+1}$ . Denote by $T^{\left(k,n\right)}\left(t_{n},t\right)$ a subtree that starts at time $t_{n}$ , for all k=0,…,n (counting from the left to the right on the tree), and by $T^{\left(k,n\right)}\left(t_{n},t_{n+1}\right)$ its segment within the time internal $\left[t_{n},t_{n+1}\right]$ . For all n=1,…,N−1 , denote by $T^{\left(\ell_{n},n\right)}\left(t_{n},t\right)$ the k -th subtree, with $k=\ell_{n}$ , on which a branching event has occurred at time $t_{n+1}$ . See example in Figure 1.

**FIGURE 1:**
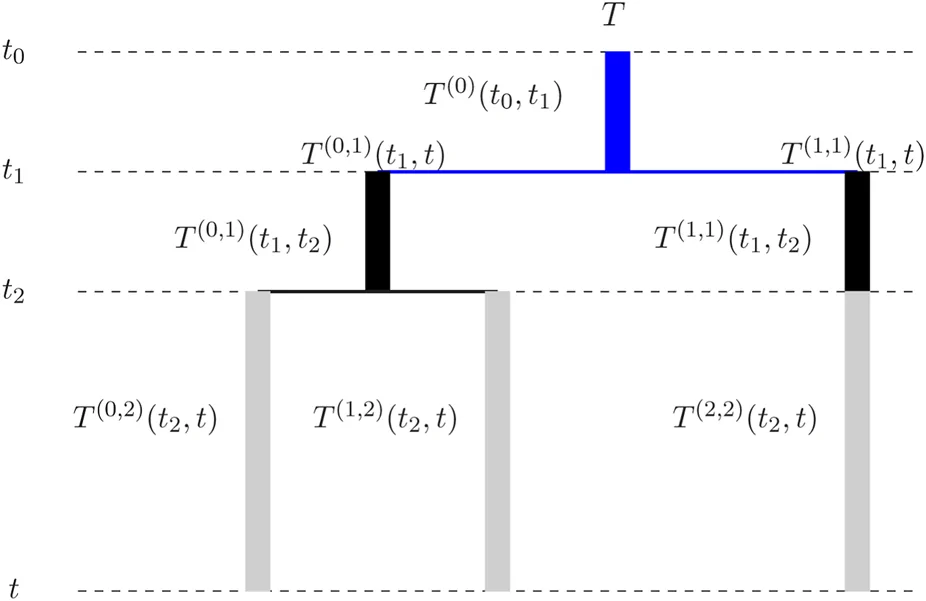
Adapted from (Soewongsono et al., 2025, Figure 13) This was done under a Creative Commons Attribution 4.0 International License (http://creativecommons.org/licenses/by/4.0/): Tree T is is decomposed into segments. Initial segment $T^{\left(0\right)}\left(t_{0},t_{1}\right)$ evolving in the time interval $\left[t_{0},t_{1}\right]$ , with the branching event at time $t_{1}$ resulting in two subtrees, $T^{\left(k,1\right)}\left(t_{1},t\right)$ for k=0,1 . The subtree $T^{\left(0,1\right)}\left(t_{1},t\right)$ is decomposed into its initial segment $T^{\left(0,1\right)}\left(t_{1},t_{2}\right)$ , and its two subtrees, $T^{\left(k,2\right)}\left(t_{2},t\right)$ for k=0,1 , and so $\ell_{1}=0$ . The subtree $T^{\left(1,1\right)}\left(t_{1},t\right)$ is decomposed into its initial segment $T^{\left(1,1\right)}\left(t_{1},t_{2}\right)$ , and the subtree $T^{\left(k,2\right)}\left(t_{2},t\right)$ with k=2 .

Suppose that the evolution of some characters within the tree is modelled using MM model (QBD/SFM) with the initial distribution $\alpha =\left[\alpha_{\left(x,i\right)}\right]$ and operator $P\left(t\right)=\left[P_{\left(x,i\right)\left(y,j\right)}\left(t\right)\right]$ which describes the evolution (x,i)→(y,j) of the MM process in time (recording probabilities for the QBDs/densities for the SFMs). For example, this could be the evolution of the gene family within the species tree (Soewongsono et al., 2025), or the evolution of traits within the species tree (Nigus et al., 2025). Then by a generalisation of the results for the QBDs and MBTs in Soewongsono et al. (2025) to MMs, the likelihood ℓ(T) of observing this tree can be evaluated with Algorithm 1. The order of expressions in this recursive algorithm is analogous to the pruning algorithm of Felsenstein (1973), Felsenstein (1981). The computations of P and f within the algorithm, require the application of the theory and algorithms in the literature of MMs (Section 2.1).

To include potential data about the states of the MM model observed within the tree modify Algorithm 1 as follows. Given state (X(t),φ(t))=s at the end of segment $T^{\left(k,N\right)}\left(t_{N},t\right)$ , simply replace $P\left(t-t_{N}\right)1$ in Equation 1 with $P\left(t-t_{N}\right)1_{s}$ , where column vector $1_{s}$ has one in row s and 0 everywhere else. Similarly, given state $\left(X\left(t_{n+1}\right),\varphi \left(t_{n+1}\right)\right)=s$ at the end of segment $T^{\left(k,n\right)}\left(t_{n},t\right)$ , replace $P\left(t_{n+1}-t_{n}\right)$ in Equation 2 with matrix $P^{\left(s\right)}\left(t_{n+1}-t_{n}\right)$ such that $P_{ij}^{\left(s\right)}\left(t_{n+1}-t_{n}\right)=P_{ij}\left(t_{n+1}-t_{n}\right)$ for j=s and 0 everywhere else.

Algorithm 1. Compute ℓ(T) (See Matlab code and numerical example in Supplementary Material).

 1: **for** k=0,…,N **do**
 2: Compute (conditional probability/density) column vector
$$\begin{array}{ll} f\left(T^{\left(k,N\right)}\left(t_{N},t\right)\right) & =P\left(t-t_{N}\right)1 \end{array}$$ (1)

   where 1 denotes a column vector.
 3: **end for**
 4: **for** n=N−1,…,1 **do**
 5:  **for** k=0,…,n **do**
 6:   Compute (conditional probability/density) column vector
$$\begin{array}{ll} f\left(T^{\left(k,n\right)}\left(t_{n},t\right)\right) & =\left\{\begin{array}{cc} P\left(t_{n+1}-t_{n}\right)f\left(T^{\left(k,n+1\right)}\left(t_{n+1},t\right)\right) & \;k<\ell_{n},\\ P\left(t_{n+1}-t_{n}\right)\left(f\left(T^{\left(k,n+1\right)}\left(t_{n+1},t\right)\right)⊙f\left(T^{\left(k+1,n+1\right)}\left(t_{n+1},t\right)\right)\right) & \;k=\ell_{n},\\ P\left(t_{n+1}-t_{n}\right)f\left(T^{\left(k+1,n+1\right)}\left(t_{n+1},t\right)\right) & \;k>\ell_{n}, \end{array}\right. \end{array}$$ (2)

   with ⊙ denoting Hadamard product (use Kronecker product if the subtrees have different initial states).
 7:  **end for**
 8: **end for**
 9: Compute likelihood, given initial distribution row vector $\alpha =\left[\alpha_{\left(x,i\right)}\right]$ of the MM process,
$$\begin{array}{ll} \ell \left(T\right) & =\alpha P\left(t_{1}-t_{0}\right)\left(f\left(T^{\left(0,1\right)}\left(t_{1},t\right)\right)⊙f\left(T^{\left(1,1\right)}\left(t_{1},t\right)\right)\right). \end{array}$$

Maximum likelihood is a well established method of phylogenetic inference (Rannala and Yang, 1996), which can be used to choose a tree based on the likelihood formula in Algorithm 1 out of a collection of candidate trees. This approach opens the door to the analysis built on the theory of MM models and the results in Soewongsono et al. (2025). For example, a compelling problem is evaluating the likelihood and the reconstruction of a tree when only the data at the tips at time t is available. The total number of alternative trees given n tips grows rapidly with n , resulting in the sequence of Euler numbers (Disanto et al., 2013). We propose to approach this problem by applying Dynamic Programming (DP) to reconstruct the tree from the tips to the root in combination with the recursion in Algorithm 1 as follows. At stage n=N+1,1 of the DP consider alternative places ( $\ell_{n}$ in (2)) where branching could have occurred and then store computations of $f\left(T^{\left(k,n\right)}\left(t_{n},t\right)\right)$ for these alternatives. Then the total number of distinct topologies built by joining through branching two subtrees out of n where m have identical topologies, is

|Sn;m|=|Sn−1;m−2|+n−m|Sn−1;m−2|+n−m2|Sn−1;m|

which results in the sequence of Euler numbers for |S(n;n)|. Approximate DP (Powell, 2009; 2011; 2016), is suitable in the optimisation of large-size DP problems, and we will explore this idea in our future works. Further, advanced MM models need to be studied in the theory of matrix-analytic methods so as to handle time-inhomogeneous evolutionary processes as the current methods do not model these problems in a sufficient manner as noted in Baake and Sumner (2025); Sumner et al. (2012). Current literature of the MM models in the theory of matrix-analytic methods largely involve models with time homogeneous parameters for the quantities required in the evaluation of the likelihood ℓ(T) with Algorithm 1. Some preliminary work on time-inhomogeneous cyclic QBDs and SFMs (Margolius, 2008; Margolius and O’Reilly, 2016; Margolius and O’Reilly, 2026) has been developed in recent years, and so there is a lot of potential for adapting these ideas to tackle problems in evolution, e.g. to include a cyclical mutation modality (Bartlett, 2024).This work is in early stages however and more needs to be done, for example, methods to tackle delay in response to environmental stimuli have not been developed in the literature of the MM models as yet. QBDs/SFMs with a multi-dimensional level variable (Aksamit et al., 2025) could be explored to analyse more complex problems such as environmentally-driven evolution of species (Soewongsono, 2023). Algorithm 1, which has been applied in the context of QBDs/MBTs in Soewongsono et al. (2025) for the evaluation of tree likelihood and reconciliation problem, could be applied to SFMs, and time-inhomogeneous MM models.

### 2.3 Conclusion

To conclude, unlocking the full application potential of the Markov modulated (MM) models to evolutionary processes can only be achieved through close multidisciplinary collaborations of applied probabilists and evolutionary biologists. As we highlighted, there exists a rich, well-developed theory of the MM models in the applied probability literature, which is not yet fully explored in the context of applications to evolution. At the same time, deep knowledge of the evolutionary processes is needed to formulate relevant theoretical problems that could be tackled with probabilistic and computational tools.

## Data Availability

The original contributions presented in the study are included in the article/supplementary material, further inquiries can be directed to the corresponding author.
